# Tracking microbial colonization in fecal microbiota transplantation experiments via genome-resolved metagenomics

**DOI:** 10.1186/s40168-017-0270-x

**Published:** 2017-05-04

**Authors:** Sonny T. M. Lee, Stacy A. Kahn, Tom O. Delmont, Alon Shaiber, Özcan C. Esen, Nathaniel A. Hubert, Hilary G. Morrison, Dionysios A. Antonopoulos, David T. Rubin, A. Murat Eren

**Affiliations:** 1 Section of Gastroenterology, Hepatology and Nutrition, Department of Medicine, University of Chicago Medicine, Chicago, IL USA; 2 Josephine Bay Paul Center for Comparative Molecular Biology and Evolution, Marine Biological Laboratory, Woods Hole, 02543 MA USA; 3 Present address: Boston Children’s Hospital, Inflammatory Bowel Disease Center, Boston, MA USA

**Keywords:** Fecal microbiota transplantation, Colonization, Metagenomics, Metagenome-assembled genomes

## Abstract

**Background:**

Fecal microbiota transplantation (FMT) is an effective treatment for recurrent *Clostridium difficile* infection and shows promise for treating other medical conditions associated with intestinal dysbioses. However, we lack a sufficient understanding of which microbial populations successfully colonize the recipient gut, and the widely used approaches to study the microbial ecology of FMT experiments fail to provide enough resolution to identify populations that are likely responsible for FMT-derived benefits.

**Methods:**

We used shotgun metagenomics together with assembly and binning strategies to reconstruct metagenome-assembled genomes (MAGs) from fecal samples of a single FMT donor. We then used metagenomic mapping to track the occurrence and distribution patterns of donor MAGs in two FMT recipients.

**Results:**

Our analyses revealed that 22% of the 92 highly complete bacterial MAGs that we identified from the donor successfully colonized and remained abundant in two recipients for at least 8 weeks. Most MAGs with a high colonization rate belonged to the order Bacteroidales. The vast majority of those that lacked evidence of colonization belonged to the order Clostridiales, and colonization success was negatively correlated with the number of genes related to sporulation. Our analysis of 151 publicly available gut metagenomes showed that the donor MAGs that colonized both recipients were prevalent, and the ones that colonized neither were rare across the participants of the Human Microbiome Project. Although our dataset showed a link between taxonomy and the colonization ability of a given MAG, we also identified MAGs that belong to the same taxon with different colonization properties, highlighting the importance of an appropriate level of resolution to explore the functional basis of colonization and to identify targets for cultivation, hypothesis generation, and testing in model systems.

**Conclusions:**

The analytical strategy adopted in our study can provide genomic insights into bacterial populations that may be critical to the efficacy of FMT due to their success in gut colonization and metabolic properties, and guide cultivation efforts to investigate mechanistic underpinnings of this procedure beyond associations.

**Electronic supplementary material:**

The online version of this article (doi:10.1186/s40168-017-0270-x) contains supplementary material, which is available to authorized users.

## Background

Fecal microbiota transplantation (FMT), the transference of fecal material from a healthy donor to a recipient, has gained recognition as an effective and relatively safe treatment for recurrent or refractory *Clostridium difficile* infection (CDI) [1–8]. Its success in treating CDI sparked interest in investigating FMT as a treatment for other medical conditions associated with intestinal dysbiosis, such as ulcerative colitis [9–11], Crohn’s disease (CD) [12–14], irritable bowel syndrome (IBS) [15, 16], and others, including metabolic syndrome [17], neurodevelopmental [18], and autoimmune disorders [19]. Despite the excitement due to its therapeutic potential, FMT also presents challenges for researchers and clinicians with potential adverse outcomes, including the transfer of infectious organisms [20] or contaminants from the environment [21, 22]. A complete understanding of FMT from a basic science perspective is still lacking, as we have yet to determine the key microbial populations that are responsible for beneficial outcomes, as well as adverse effects.

Recent advances in high-throughput sequencing technologies, molecular approaches, and computation have dramatically increased our ability to investigate the ecology of microbial populations. Utilization of these advances at a proper level of resolution can lead to a better mechanistic understanding of FMT and identify new therapeutic opportunities or address potential risks. Most current studies on FMT use amplicons from marker genes, such as the 16S ribosomal RNA gene, to characterize the composition of microbial communities [23–26]. While providing valuable insights into the broad characteristics of FMTs, amplicons from the 16S ribosomal RNA gene do not offer the resolution to effectively identify populations that colonize recipients [27]. Other studies use shotgun metagenomics to annotate short reads and map them to reference genomes in order to track changes in the functional potential or membership in the gut microbial communities of recipients [28–30]. In a recent study, Li et al. [30] demonstrated the coexistence of donors and recipients’ gut microbes 3 months after FMT by mapping short metagenomic reads to reference genomes. Although this approach provides more information than marker gene amplicons alone, it is subject to the limitations and biases of reference genomic databases, is unable to characterize populations that do not have closely related culture representatives, and does not provide direct access to the genomic context of relevant populations for more targeted follow-up studies.

Metagenomic assembly and binning [31, 32] is an alternative approach to characterizing microbial communities through marker gene amplicons or reference genomes. Here, we used the state-of-the-art metagenomic assembly and binning strategies to reconstruct microbial population genomes directly from a single FMT donor and tracked the occurrence of resulting metagenome-assembled genomes (MAGs) in two FMT recipients up to 8 weeks.

## Methods

### Sample collection, preparation, and sequencing

We collected a total of 10 fecal samples; four samples from a single donor “D” (a 30-year-old male) and three samples from each of the two recipients “R01” (a 23-year-old male) and “R02” (a 32-year-old female) before and after FMT. Recipient samples originated from time points pre-FMT, 4 weeks after FMT, and 8 weeks after FMT, while four samples from the donor were collected on four separate days 2 weeks prior to the transplantation. All fecal samples were handled under anaerobic conditions prior to transplantation, and the recipients had no genetic relationship to the donor. Through a single colonoscopy for each recipient, the donor sample DS 01 was transferred to R01, and the donor sample DS 04 was transferred to R02. All samples were stored at −80 °C until DNA extraction. We extracted the genomic DNA from frozen samples according to the centrifugation protocol outlined in MoBio PowerSoil kit with the following modifications: cell lysis was performed using a GenoGrinder to physically lyse the samples in the MoBio Bead Plates and Solution (5–10 min). After final precipitation, the DNA samples were resuspended in TE buffer and stored at −20 °C until further analysis. We prepared our shotgun metagenomic libraries with OVATION Ultralow protocol (NuGen) and used an Illumina NextSeq 500 platform to generate 2 × 150 nt paired-end sequencing reads.

### Metagenomic assembly and binning

We removed the low-quality reads from the raw sequencing results using the program “iu-filter-quality-minoche” in illumina-utils [33] (available from https://github.com/merenlab/illumina-utils) according to Minoche et al. [34]. We then co-assembled reads from the donor samples using MEGAHIT v1.0.6 [35], used Centrifuge v1.0.2-beta [36] to remove contigs that matched to human genome, and mapped short reads from each recipient and donor sample to the remaining contigs using Bowtie2 v2.0.5 [37]. We then used anvi’o v2.3.1 (available from http://merenlab.org/software/anvio) to profile mapping results, finalize genomic bins, and visualize results following the workflow outlined in Eren et al. [38]. Briefly, (1) the program “anvi-gen-contigs-database” profiled our contigs using Prodigal v2.6.3 [39] with default settings to identify open reading frames and HMMER [40] to identify matching genes in our contigs to bacterial [41] and archaeal [42] single-copy core gene collections, (2) “anvi-init-bam” converted mapping results into BAM files, (3) “anvi-profile” processed each BAM file to estimate the coverage and detection statistics of each contig using samtools [43], and finally, (4) “anvi-merge” combined profiles from each sample to create a merged anvi’o profile for our dataset. We used “anvi-cluster-with-concoct” for the initial binning of contigs using CONCOCT [44] by constraining the number of clusters to 10 (“--num-clusters 10”) to minimize the “fragmentation error” (where multiple bins describe one population). We then interactively refined each CONCOCT bin that exhibit “conflation error” (where one bin describes multiple populations) using the program “anvi-refine” based on tetra-nucleotide frequency, taxonomy, mean coverage, and completion and redundancy estimates based on bacterial and archaeal single-copy genes. We classified a given genome bin as a “metagenome-assembled genome” (MAG) if it was more than 70% complete or larger than 2 Mbp, and its redundancy was estimated to be less than 10%. We used “anvi-interactive” to visualize the distribution of our bins across samples and “anvi-summarize” to generate static HTML output for binning results. Besides anvi’o, we also used CheckM v1.0.7 [45] to assess the completion and contamination of our bins.

### Taxonomic and functional annotation of MAGs

We employed multiple approaches to infer taxonomy. Besides the taxonomic annotations reported by CheckM, we searched amino acid sequences for the RecA gene for each MAG in the National Center for Biotechnology Information (NCBI) databases. We also used Phylosift v1.0.1 [46] to determine the phylogenomic relationships between our MAGs and a collection of 1758 reference genomes (with no redundancy at the species level) that we acquired from the Ensembl database [47] (Additional file 1: Table S3). Briefly, Phylosift (1) identifies a set of 37 marker gene families in each genome, (2) concatenates the alignment of each marker gene family across genomes, and (3) computes a phylogenomic tree from the concatenated alignment using FastTree [48]. Lastly, we used FigTree v1.4.3 (http://tree.bio.ed.ac.uk/software/figtree/) to finalize the phylogenomic tree for publication. We used RAST [49] to ascribe functions to our MAGs.

### Criteria for detection and colonization of MAGs in the recipients

For each genome bin, anvi’o reports the percentage of nucleotide positions in all contigs that are covered by at least one short read based on mapping results. This statistic gives an estimate of “detection” regardless of the coverage of a given genome bin. We required the detection statistic of a genome bin to be at least 25% to consider it “detected” in a given sample. This prevented inflated detection rates due to non-specific mapping, which is not uncommon due to relatively well-conserved genes across gut populations. Finally, we conservatively decided that a MAG was transferred from the donor and colonized a given recipient successfully only if (1) it was detected in both samples that were collected from the recipient at 4 and 8 weeks after the FMT and (2) it was not detected in the pre-FMT sample from the same recipient.

### The use of HMP metagenomes

We used 151 Human Microbiome Project (HMP) gut metagenomes [50] to estimate the detection of our MAGs and to compare the taxonomic profiles of our metagenomes in the context of the HMP participants. To estimate detection, we mapped HMP metagenomes to our MAGs using Bowtie2 with default parameters and considered a MAG to be detected in a given HMP metagenome when its level of detection surpassed 25%. We also annotated our metagenomes and HMP gut metagenomes using MetaPhlAn2 [51]. Additionally, to estimate the contribution of donor MAGs to recipient taxonomic profiles, we used the 60 and 83 MAGs that were not detected pre-FMT in samples of R01 and R02 to recruit and remove reads from the post-FMT R01 and R02 metagenomes. MetaphlAn2 estimated the taxonomical profiles of the remaining reads. Additional file 1: Table S3 reports taxonomic annotations.

### Statistical analyses

We performed cluster analyses on distribution profiles of MAGs using the R library vegan with Bray-Curtis distances of normalized values. We used the PERMANOVA (R adonis vegan) [52] test to measure the degree of similarity of the bacterial communities between the samples in the study. We further used similarity index (SIMPER) analysis to identify the taxa that contributed the highest dissimilarity between the samples. We classified the MAGs into four main groups based on their colonization characteristics in the recipients. We then performed a Wilcoxon signed-rank test (STAMP) [53] with Benjamini-Hochberg FDR (false discovery rate) correction for multiple tests on the total-sum normalized data to ascertain any significant differences in the functional potential between the groups and carried out canonical correspondence analysis based on functional potential and the MAGs’ colonization characteristics.

## Results

**Fig. 1:**
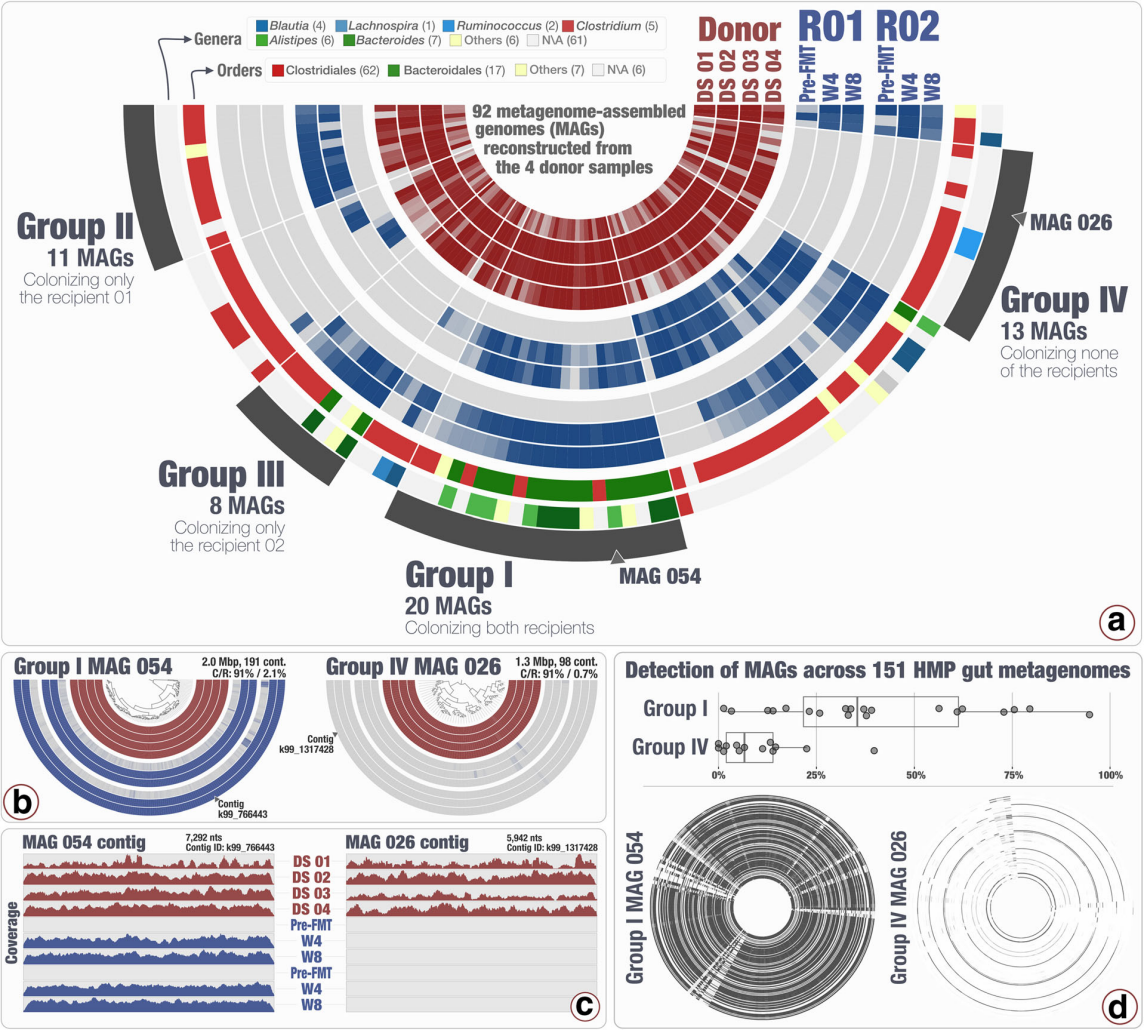
Distribution of MAGs across samples and HMP metagenomes. **a** The 92 MAGs and their level of detection in four donor samples (*four inner circles*) as well as two recipients (R01 and R02) before FMT (pre-FMT), 4 weeks after FMT (W4), and 8 weeks after FMT (W8). *Rectangles* with *red* and *blue* colors in donor and recipient layers indicate the level of detection of a given MAG in a given sample. The outermost two layers display the genus- and order-level taxonomy for each MAG. Selections in **a** represent four groups of MAGs based on their distribution patterns: group I with 20 MAGs that colonized both recipients, group II with 11 MAGs that colonized only R01, group III with 8 MAGs that colonized only R02, and finally, group IV with 13 MAGs that colonized neither recipient. **b** The detection for each contig in two example MAGs summarized to a single detection value in **a**. **c** The coverage of each nucleotide position in two example contigs from the MAGs displayed in **b**. **d** The prevalence of MAGs in groups I and IV across 151 HMP gut metagenomes and detection of MAG 54 (group I) and MAG 26 (group IV) in HMP gut metagenomes as two examples

The shotgun sequencing of genomic DNA from 10 fecal samples resulted in a total of 269,144,211 quality-filtered 2 × 150 paired-end metagenomic reads (Additional file 2: Table S1). By co-assembling the donor samples, which corresponded to 115,037,928 of the quality-filtered reads, we recovered 51,063 contigs that were longer than 2.5 kbp and organized them into 444 genomic bins comprising a total of 442.64 Mbp at various levels of completion (Additional file 2: Table S1, Additional file 3: Figure S1). Using completion and size criteria, we designated 92 of our genomic bins as metagenome-assembled genomes (MAGs) (Fig. 1, Additional file 2: Table S1). Four major patterns emerged from the distribution of MAGs across individuals: MAGs that colonized both recipients R01 and R02 (group I, *n* = 20), MAGs that colonized only R01 (group II, *n* = 11), only R02 (group III, *n* = 8) and MAGs that did not colonize either of the recipients (group IV, *n* = 13) (Fig. 1). We found no correlation between the abundances of MAGs in donor samples and their success at colonizing recipients (ANOVA, *F* = 0.717, *p* = 0.543). Additional file 2: Table S1 reports the detection and mean coverage statistics for each MAG in each group. Both of our recipients had mild/moderate ulcerative colitis (R01 had proctitis and R02 had left-sided/distal colitis), and both of them showed a decrease in intestinal inflammation after FMT. R01 had an initial fecal calprotectin count of 26 prior to FMT, which decreased to <17 at week 4 (no data was collected at week 8), and R02’s fecal calprotectin count decreased from 947 (pre-FMT) to 68 and 34 in week 4 (W4) and week 8 (W8), respectively.

The taxonomy of 14 of the 20 MAGs that colonized both recipients resolved to the order Bacteroidales (Fig. 1). Besides Bacteroidales, group I also included five MAGs that were classified as order Clostridiales and one MAG as Coriobacteriales. CheckM partitioned the group I MAGs into *Bacteroides* (*n* = 5), *Alistipes* (*n* = 5), *Odoribacter* (*n* = 1), *Paraprevotella* (*n* = 1), and *Barnesiella* (*n* = 1). Seven MAGs were not assigned to a specific genus in this group. In contrast to the Bacteroidales-dominated group I, 10 of the 13 MAGs that did not colonize recipients (group IV) resolved to the order Clostridiales. The remaining three MAGs were not assigned any taxonomy at the order level. The only genus-level annotation for the MAGs in group IV was *Ruminococcus* (*n* = 2). Overall, CheckM did not assign any genus-level taxonomy to 18 of the 33 MAGs in group I and group IV. MAGs that colonized only one of the two recipients did not show a consistent taxonomic signal: while 9 of 11 MAGs that colonized only R01 (group II) were assigned to the order Clostridiales, only 4 of 8 MAGs that only colonized R02 (group III) were assigned to that order (Fig. 1, Additional file 2: Table S1). The remaining MAGs in group III were assigned to orders Bacteroidales (*n* = 2) and Burkholderiales (*n* = 1), and one of them did not resolve to any order-level taxon (*n* = 1). We also employed a phylogenomic approach to investigate the validity of the taxonomy of our MAGs with respect to reference genomes (Additional file 4: Figure S2). All order-level taxonomic annotations by CheckM were consistent with the phylogenomic placement of our MAGs. We resolved inconsistencies at the genus-level by removing annotations that differed between approaches.

We used the Human Microbiome Project gut metagenomes to investigate whether the differential colonization outcomes we observed for donor MAGs were representative of their occurrence in healthy individuals. Our analysis of 151 publicly available gut metagenomes showed that the donor MAGs in group I that colonized both recipients were more prevalent, and the ones in group IV that colonized neither of the recipients were more rare across the participants of the HMP (ANOVA, *F* = 10.04, *p* < 0.001; Fig. 1). In fact, while the group I MAGs occurred in 40.63% (±26.67%) of the individuals, the group IV MAGs showed more sporadic patterns as they were detected in only 10.39(±5.69%) of the HMP metagenomes (Fig. 1, Additional file 1: Table S3, Additional file 5: Figure S3). These results suggest that the relevance of the donor MAGs we recovered here are not necessarily limited to our study and represent a subset of populations with patterns of high- and low-colonization success.

**Fig. 2:**
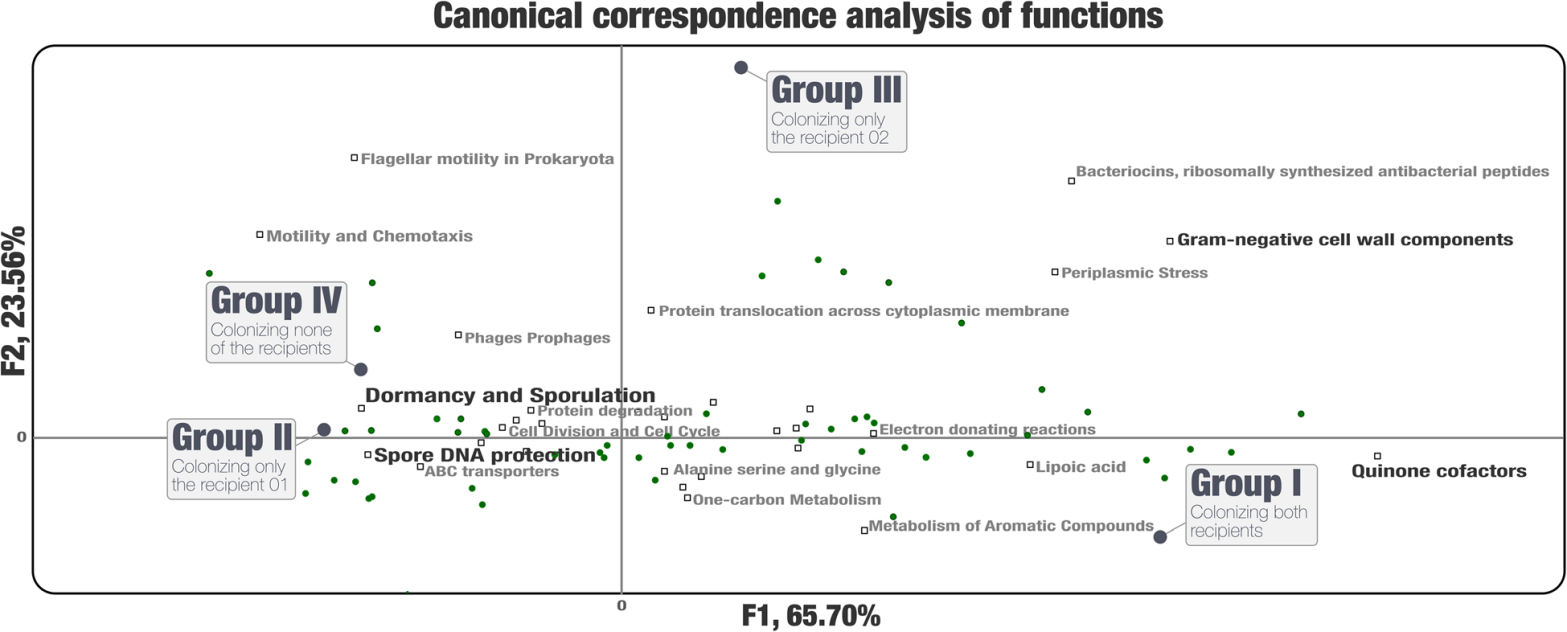
Canonical correspondence analysis of functions in four groups of MAGs. The 39 significant functional subcategories are shown

We then investigated whether there was a link between the functional potential of MAGs and their success of colonization. The canonical correspondence analysis (CCA) of 500 functions and 107 sub-systems (Additional file 6: Table S2) revealed that the group I MAGs possessed a higher relative abundance of genes coding for quinone cofactors, along with functions involving lipoic acid synthesis and metabolism of aromatic compounds. In contrast, the group IV MAGs carried higher number of genes related to dormancy and sporulation, spore DNA protection, and motility and chemotaxis (pseudo-F = 2.156, *p <* 0.0001). Interestingly, MAGs that were transferred to only R01 (group II) also carried higher numbers of genes for dormancy and sporulation and spore DNA protection (Fig. 2, Additional file 6: Table S2). The functional similarity between group II and group IV MAGs was also mirrored with their detection rate across the HMP metagenomes (Additional file 1: Table S3, Additional file 5: Figure S3). This suggests that despite strong signal, the functional potential is not a robust predictor of colonization success and individuals may respond differently to the same donor.

**Fig. 3:**
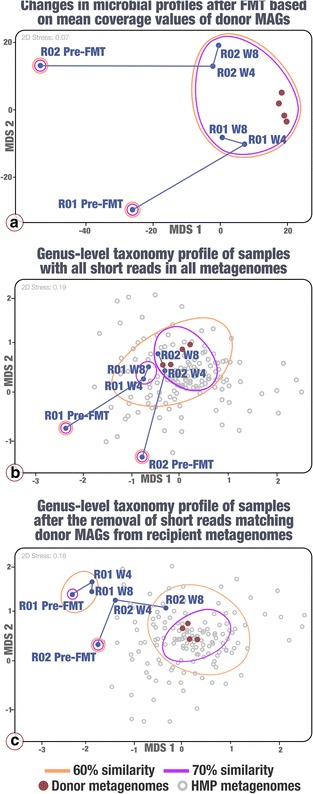
Similarity between donor and recipient samples before and after FMT. Non-metric multidimensional scaling based on mean coverage of 92 MAGs and based on microbial community profiles from this study and 151 HMP metagenomes at the genus level of short reads annotated by MetaPhlAn. Clustering employed average linkage with Bray-Curtis similarity index on square-root normalized values. Labels represent the recipients (R01, R02) before FMT (pre-FMT), 4 weeks (W4) and 8 weeks after FMT (W8). *Gray circles* represent HMP metagenomes. Panel **a** displays changes in recipient microbial community profiles after FMT based on coverage values of donor MAGs. Panel **b** displays the organization of samples based on the genus-level taxonomy of all short metagenomic reads in each sample. In contrast, Panel **c** displays the genus organization of samples based on the genus-level taxonomy of only short metagenomic reads that do not match donor MAGs

We also investigated to what extent the recipients became donor-like after the FMT. As expected, the analysis based on the mean coverage of donor MAGs suggested an increased similarity between the donor and recipients after the FMT (Fig. 3a). Mapping metagenomic short reads to donor MAGs offers a limited insight into the recipient microbial populations. To minimize any potential biases, we also generated genus-level taxonomic profiles for donor and recipient samples by annotating all metagenomic short reads. This strategy also allowed us to compare our donor, as well as our recipients before and after FMT, to the larger context of the HMP cohort (Fig. 3b). This analysis suggested that the donor samples showed no discernible differences from the HMP cohort (PERMANOVA, pseudo-F = 1.489, *p* = 0.110). In contrast, the pre-FMT samples of both recipients differed significantly from the HMP cohort and from the donor samples (PERMANOVA, pseudo-F = 4.470, *p* = 0.001). However, the recipient samples after FMT no longer differed from the HMP cohort (PERMANOVA, pseudo-F = 1.395, *p* = 0.168) (Fig. 3b). Based on the taxonomic profiles from short metagenomic reads, both recipients were more than 60% similar to the donor microbiota after FMT. Similarity percentage analysis (SIMPER) of the taxonomic profiles suggested that the two recipients were 79.60% similar after FMT and that *Bacteroides* was responsible for the largest fraction (32.42%) of difference between recipient samples of pre-FMT and 4 weeks after FMT. There were no significant changes in the recipients’ taxonomic profiles between W4 and W8 after FMT (PERMANOVA, pseudo-F = 0.221, *p* = 0.631). These results show that our recipients, who were outliers to the HMP cohort, became more like the HMP cohort following FMT. Yet, one important question remains: to what extent this convergence is due to donor MAGs and not due to the changing abundance of the initially rare members of the recipient microbial populations? To investigate this, we first identified donor MAGs that were not detected in a given recipient’s pre-FMT metagenome and removed short reads from the recipient’s W4 and W8 metagenomes after FMT. We then characterized the taxonomic profiles with the remaining reads (Fig. 3c). Without reads matching to donor MAGs, the taxonomic profiles for the samples collected after FMT remained close to the pre-FMT status, and dissimilar to both the donor and HMP metagenomes, suggesting a more important contribution of the donor populations to the convergence rather than the emergence of initially rare recipient populations. This was particularly apparent for R01, for which the taxonomic profiles before and after FMT at W8 were highly dissimilar (*R* ^2^ of 0.122) yet became more alike after the subtraction of reads matching to donor MAGs (*R* ^2^ of 0.634). Taking into account the fact that 92 MAGs are only a subset of the 444 bins we initially recovered from the donor, we tested whether these trends remained similar when all 444 metagenomic bins were used for removal of short reads and confirmed that the outcome did not change (Additional file 7: Figure S4).

## Discussion

Here, we demonstrate that shotgun metagenomics can facilitate the tracking of bacterial populations in FMT experiments by linking their colonization trends to genomic contexts through metagenome-assembled genomes (MAGs). In our study, the relative abundance of MAGs in donor metagenomes did not predict whether they would colonize the FMT recipients or not. However, their success in colonizing the recipients mirrored their occurrence in the large cohort of the Human Microbiome Project (HMP): the MAGs that colonized both of our recipients were also found in many of the participants of the HMP, and the ones that colonized neither of our recipients were missing in many. Besides suggesting future directions to investigate the ecological and functional basis of gut colonization, this observation suggest that perhaps large-scale metagenomic surveys can be useful to predict the colonization properties of bacterial populations rapidly.

Previous studies reported an increase in the relative abundance of *Alistipes* [23, 24, 54–56] and *Bacteroides* populations after FMT experiments [23–26, 30]. The success of the order Bacteroidales was also striking in our dataset: 14 of the 20 group I Bacteroidales MAGs we identified in the donor successfully colonized both recipient guts. Although the taxonomic signal was relatively strong, our results also showed that taxonomy is not the sole predictor of transfer, as MAGs that resolved to the same genera (i.e., *Alistipes*, *Bacteroides*, and *Clostridium*) showed different colonization properties. In addition, taxonomic annotation of a large fraction of MAGs in our study did not resolve to a genus name, which suggests that bacterial populations that have not yet been characterized in culture collections may be playing important roles in FMT treatments.

Although a substantial number of studies report successful medical outcomes of FMT experiments [3, 7, 57, 58], a complete understanding of this procedure from the perspective of microbial ecology is still lacking. Studying FMT as an ecological event and the identification of its key components that facilitate the procedure’s success as a treatment for intestinal disorders require a detailed characterization of the transferred microbial populations at an appropriate level of resolution. MAGs reconstructed directly from donor samples can provide enough resolution to guide cultivation efforts. A recent effort by Vineis et al. [59] demonstrated this principle by first identifying bacterial populations of interest using MAGs reconstructed from a gut metagenome and then using the genomic context of those MAGs to screen culture experiments from the same gut sample to bring the target population to the bench. A similar approach in the context of FMTs can provide opportunities to design experiments to explore the functional basis of colonization in controlled systems.

The complete transfer of fecal matter between individuals comes with various risks. For instance, a recent meta-analysis of 50 peer-reviewed FMT case reports reported 38 potentially transfer-related adverse effects in FMT patients in 35 studies, including fever, sore throat, vomiting, abdominal pain, bowel perforation, rhinorrhea, transient relapse of UC and CDI, and in one case, death, due to temporary systemic immune response to the applied bacteria [60]. Besides bacteria, FMT can transfer viruses, archaea, and fungi, as well as other agents of the donor host such as colonocytes [61], which may affect the recipient’s biology in unexpected ways. A more complete understanding of the microbial ecology of FMTs could identify precisely what needs to be transferred so that recipients benefit from the positive outcomes of FMT without incurring medical risks from uncharacterized biological material.

A recent study by Khanna et al. [62] reported high rates of success with the treatment of patients with primary *Clostridium difficile* infection (CDI) using an investigational oral microbiome therapeutic, SER-109, which contains bacterial spores enriched and purified from healthy donors. However, Seres Therapeutics announced more recently that interim findings from the mid-stage clinical study of SER-109 failed to meet their primary goal of reducing the risk of recurrence for up to 8 weeks [63]. In our study, the MAGs that failed to colonize any of the recipients were significantly enriched for spore-formation genes, and they also showed a very sporadic distribution across the HMP cohort. Interestingly, Nayfach et al. [64] recently made a similar observation regarding the transmission of bacteria and sporulation in a different system: vertical transmission of bacteria between mothers and their infants. Bacterial populations with high-vertical transmission rates had lower number of genes related to sporulation [64]. These observations suggest that excluding non-spore forming bacteria may decrease the efficacy of FMT therapies due to limited colonization efficiency, and deeper insights into the functional basis of microbial colonization require further study.

## Conclusions

Using bacterial populations associated with positive health outcomes and that harbor high colonization properties may result in more effective therapies compared to cleansing all but spore-forming bacteria to avoid the transfer of pathogens in FMT experiments. The analytical strategy adopted in our study can provide genomic insights into bacterial populations that may be critical to the efficacy of FMT due to their success in gut colonization and metabolic properties and guide cultivation efforts to investigate mechanistic underpinnings of this procedure beyond associations.

## Additional files

Additional file 1: Table S3. Phylogenomic relationships between our MAGs and a collection of 1758 reference genomes. (XLSX 775 kb)

Additional file 2: Table S1. Additional information for samples, metagenomic bins, and metagenome-assembled genomes in our study. (XLSX 180 kb)

Additional file 3: Figure S1. The distribution of all 444 donor bins across samples. Yellow markings represent the 92 MAGs. (PNG 7138 kb)

Additional file 4: Figure S2. Phylogenomic analysis of 92 MAGs in the context of 1758 gold standard genomes. Squares and triangles next to MAGs indicate the agreements (green) and disagreements (red) between the phylogenomic analysis and CheckM results. The blank ones indicate that we did not assign any taxonomy for a given MAG at the specified level. (PNG 1729 kb)

Additional file 5: Figure S3. Detection of the donor MAGs across 151 HMP metagenomes. (PNG 983 kb)

Additional file 6: Table S2. The canonical correspondence analysis (CCA) of 500 functions and 107 sub-systems. (XLSX 240 kb)

Additional file 7: Figure S4. Non-metric multidimensional scaling based on microbial genus-level taxonomy of 4 donors, 6 recipients, and 151 HMP metagenomes after the removal of short reads matching to donor bins from recipient metagenomes. (PNG 2235 kb)

## Abbreviations

FMT: Fecal microbiota transplantation
MAG: Metagenome-assembled genomes
