# Supplementary figures and images for: Tracking microbial colonization in fecal microbiota transplantation experiments via genome-resolved metagenomics
Source: Microbiome. 2017 May 4;5:50. doi: 10.1186/s40168-017-0270-x (PMC5418705; doi:10.1186/s40168-017-0270-x)

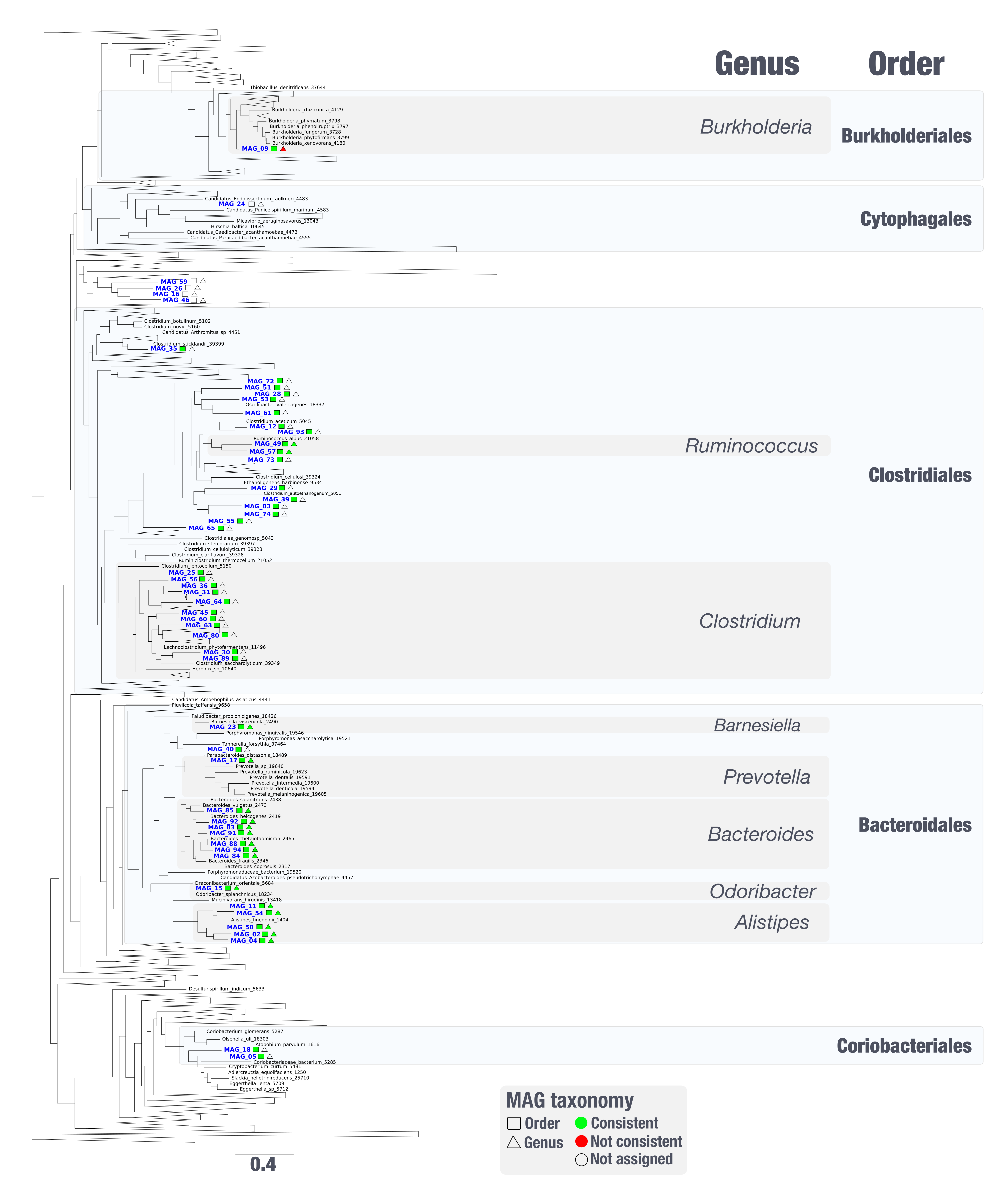

Supplement: Supplementary file 4 — Phylogenomic analysis of 92 MAGs in the context of 1758 gold standard genomes. Squares and triangles next to MAGs indicate the agreements (green) and disagreements (red) between the phylogenomic analysis and CheckM results. The blank ones indicate that we did not assign any taxonomy for a given MAG at the specified level. (PNG 1729 kb) [file 40168_2017_270_MOESM4_ESM.png]

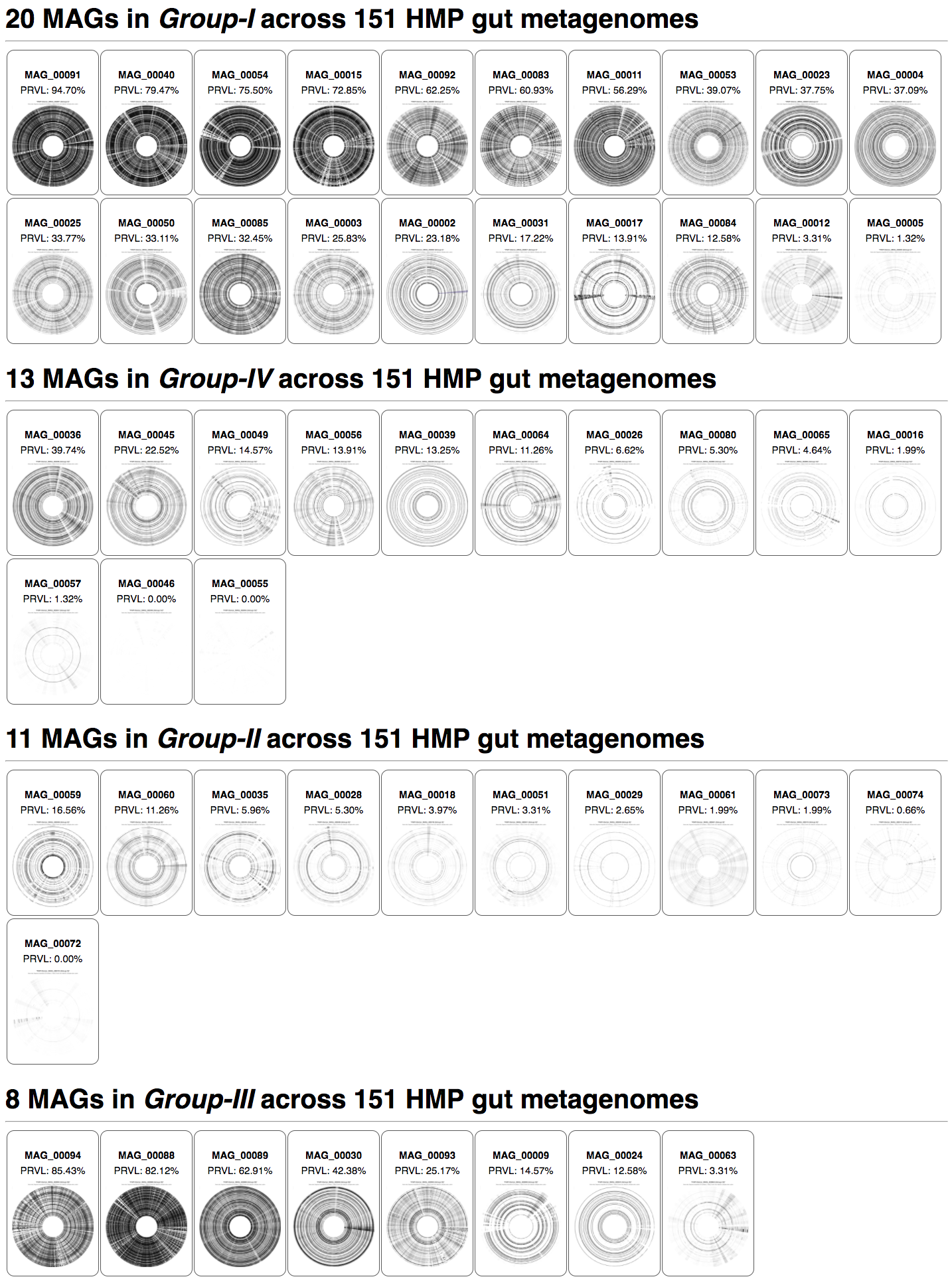

Supplement: Supplementary file 5 — Detection of the donor MAGs across 151 HMP metagenomes. (PNG 983 kb) [file 40168_2017_270_MOESM5_ESM.png]
